# Comment on “Background Ionizing Radiation and the Risk of Childhood Cancer: A Census-Based Nationwide Cohort Study”

**DOI:** 10.1289/ehp.1510111

**Published:** 2015-08-01

**Authors:** Jeffry A. Siegel, Bill Sacks, retired, Ludwig E. Feinendegen, James S. Welsh, Krzysztof W. Fornalski, Mark Miller, Jeffrey Mahn, retired, Leo Gomez, retired, Michael G. Stabin, Patricia Lewis, Vincent J. Esposito, Andrzej Strupczewski, Charles W. Pennington, Jerry M. Cuttler, Chary Rangacharyulu, Chris Davey, Shizuyo Sutou

**Affiliations:** 1 Nuclear Physics Enterprises, Marlton, New Jersey, USA; 2 U.S. Food and Drug Administration, Washington, DC, USA; 3 Heinrich-Heine-University, Dusseldorf, Germany; 4 Stritch School of Medicine, Loyola University, Chicago, Illinois, USA; 5 Polish Nuclear Society, Warsaw, Poland; 6 Sandia National Laboratories, Albuquerque, New Mexico, USA; 7 Vanderbilt University, Nashville, Tennessee, USA; 8 Free Enterprise Radon Health Mine, Boulder, Montana, USA; 9 University of Pittsburgh, Pittsburgh, Pennsylvania, USA; 10 National Centre for Nuclear Research, Warsaw, Poland; 11 NAC International, Norcross, Georgia, USA; 12 Cuttler & Associates, Mississauga, Ontario, Canada; 13 University of Saskatchewan, Saskatoon, Saskatchewan, Canada; 14 King Abdullah University of Science and Technology, Thuwal, Jeddah, Saudi Arabia; 15 Shujitsu University, Okayama, Japan

We read with interest the article by Spycher et al. The authors claim their results suggest an increased risk of cancer among children exposed to external dose rates of background ionizing radiation of ≥ 200 nSv/h, compared with those exposed to < 100 nSv/h. However, all that the data show is a positive correlation rather than a causal result, which the word “risk” implies. Besides, these dose rates correspond to annual exposure levels of approximately 1.8 and 0.9 mSv, respectively. Considering that the average natural background exposure rate in the world is on the order of 2 mSv annually, with regions that range up to as much as 260 mSv (Ghiassi-Nejad et al. 2002), these are very low doses.

Importantly, the background exposure rates were based not on actual measurements at children’s homes but on a geographic model. The authors noted they could not “exclude biases due to inaccurate exposure measurement.” It comes as no surprise, therefore, that the various hazard ratios are for the most part extremely low, and most of the 95% confidence intervals include the value of unity. Essentially, for children putatively exposed to a background dose rate exceeding 200 nSv/h, only the confidence intervals for all cancers, leukemias, and acute lymphoblastic leukemias exclude unity.

This, taken seriously, would suggest a markedly increased cancer risk for these children, based on those exposure rates, but only if one begins by assuming that these levels of radiation contribute to producing cancers. There are numerous studies that show that such levels, in fact, elicit protective biological responses that lower the risk of cancer (Doss and Little 2014; Luckey 2008). Furthermore, given the very low attributed exposure rates and the imprecision in the actual exposure estimates, it is more likely than not that this increased childhood cancer occurrence is due to causes other than the background radiation exposure.

For example, it is of interest that those children experiencing the highest estimated background dose rates are those who live in rural areas and in neighborhoods of lowest socioeconomic status. The authors state that adjustments were made for these two confounding factors, but since not much detail was provided regarding the adjustments made, the adequacy of the removal of these factors as causative contributions cannot be independently verified. Nevertheless, it is far more likely that these two factors are more important causes of childhood disease than the extremely low background exposures involved.

Moreover, if it were true that exposure rates above 200 nSv/h, low though they be, were to somehow result in such a markedly increased cancer risk for children, the only reasonable governmental policy action would be to evacuate those children living in rural areas and poor neighborhoods, and relocate them to areas with lower radiation exposure in order to save lives. Failure to act in this manner would leave the government liable for allowing its younger citizens to die at an alarming rate. Studies like this cannot be taken seriously without such public health policy implications being likewise taken seriously.
